# “Left ventricular lipoma….. a rare case”, case report

**DOI:** 10.1186/s13019-020-01122-1

**Published:** 2020-05-12

**Authors:** Fahad Shamsi, Gurjyot Bajwa, Hussam Ghalib

**Affiliations:** Heart & Vascular Institute, Cleveland Clinic Abu Dhabi, Abu Dhabi, United Arab Emirates

**Keywords:** Cardiac tumor, Lipoma, Left ventricle

## Abstract

**Background:**

A cardiac lipoma is a rare primary cardiac tumor. They are usually asymptomatic and carry a good prognosis. Cardiac Magnetic Resonance Imaging (CMR) is the confirmatory investigation of choice.

**Case presentation:**

We present a case of left ventricular lipoma in an asymptomatic patient, which was successfully treated with surgical resection.

**Conclusion:**

Cardiac lipomas are rare and are usually benign. There is no guideline on the management of cardiac lipomas and treatment is individualized.

## Background

Primary cardiac tumors are rare, accounting for less than 5% of all cardiac tumors [1]. Benign tumors comprise more than 75% of primary cardiac tumors, with myxomas being the most common, followed by papillary fibro-elastomas and lipomas. Cardiac lipomas are very rare. They constitute 2–8% of all benign cardiac tumor [2]. Most lipomas are asymptomatic and portend a favorable prognosis, but some are large enough to cause obstruction and resultant symptoms of dizziness, dyspnea and syncope. Conduction abnormalities and sudden cardiac death can also occur, but the true incidence is unknown**XXX**. We present the case of a left ventricular lipoma in an asymptomatic patient, which was diagnosed on routine screening echocardiography.

## Case presentation

**Fig. 1:**
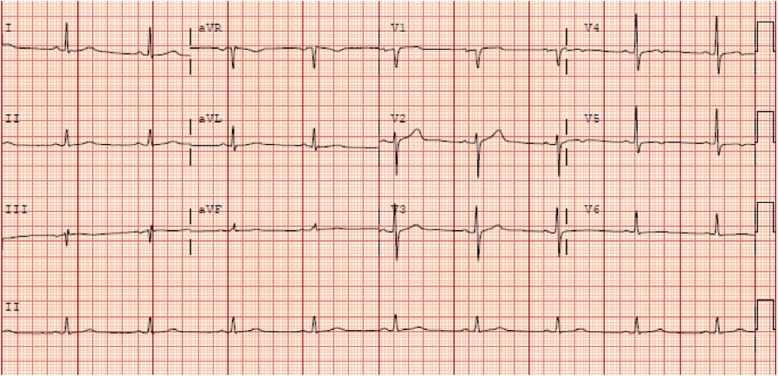
ECG showing non-specific T wave abnormalities in leads V4-V6

**Fig. 2:**
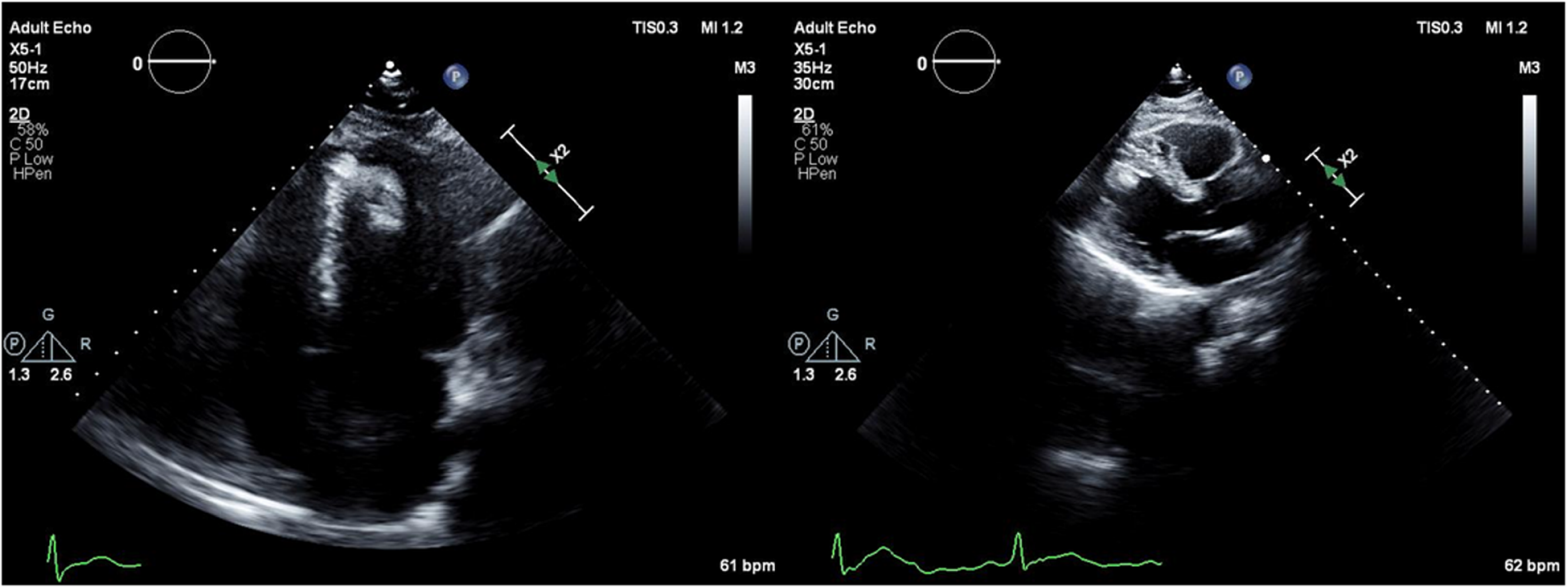
Transthoracic echocardiogram (long and short axis view) showing a pendunculated lobular mass

A 57 year old male patient with diabetes mellitus, hypertension, and hyperlipidemia underwent routine screening ECG and echocardiography as part of his annual health review. He was completely asymptomatic, with no cardiovascular symptoms. His Electrocardiogram (ECG) showed non-specific T wave abnormalities in the lateral leads (Fig. 1). His transthoracic echocardiogram showed a highly-mobile pedunculated lobular mass of 3 × 1.8 cm (Fig. 2), attached to apical septum of the left ventricle. Left ventricular (LV) wall motion and systolic function were normal.

**Fig. 3:**
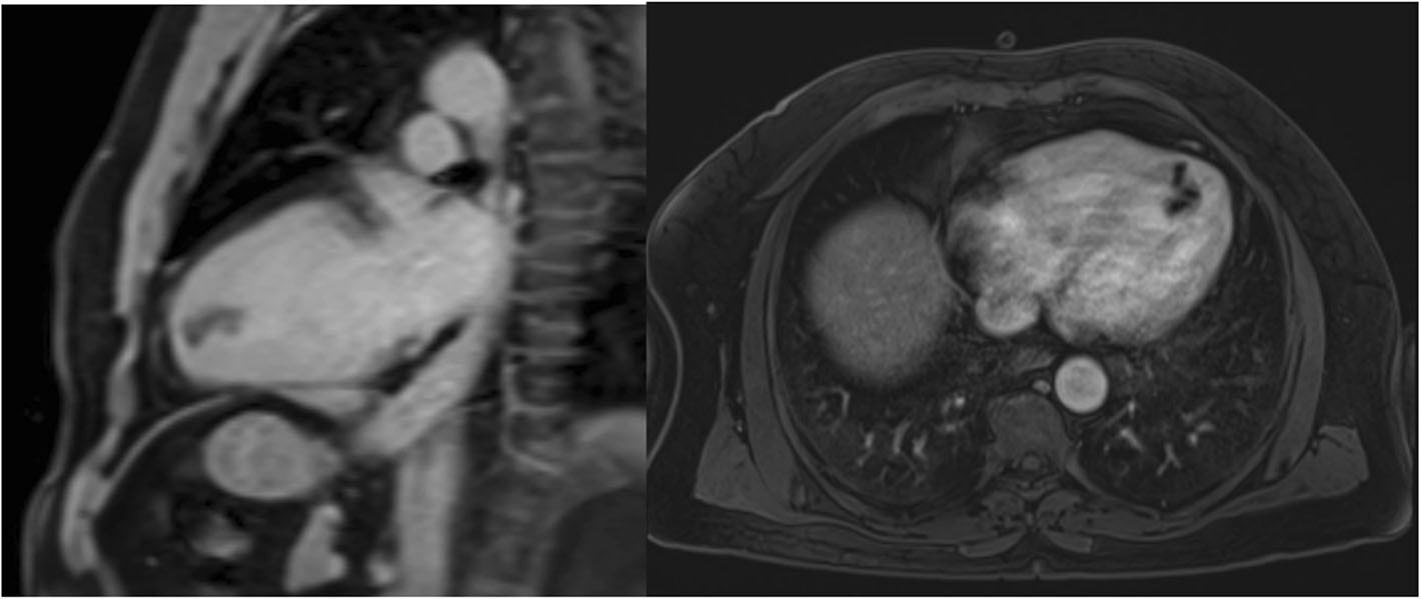
CMR demonstrating a pedunculated mobile non- enhancing mass within the LV apex, which sits on a stalk that extends into a deep crypt within the apical septum

He was admitted to his local hospital, and transferred to our facility for further work-up and management. The differential diagnoses that were entertained were LV thrombus and LV mass. CMR was performed for further tissue characterization. CMR demonstrated a pedunculated mobile non-enhancing 2 × 1.3 cm mass within the LV apex, which sits on a stalk that extends into a deep crypt within the apical septum (Fig. 3). T2 imaging demonstrated complete signal dropout within the entire mass, which was consistent with hemosiderin (i.e. thrombus). The case and images were discussed in a multi-disciplinary heart team meeting, and the final consensus was consider it an LV thrombus, treat him with warfarin-based systemic anticoagulation, and repeat imaging to show eventual resolution. Repeat echocardiography and CMR after 8 weeks of anticoagulation showed no change in the size or characteristics of the LV abnormality. At that point, the case was re-discussed in a multidisciplinary heart team meeting, and the consensus was that the mass was likely an LV myxoma. Surgical resection was recommended. Pre-operative invasive coronary angiography demonstrated non-obstructive stenosis in the proximal left anterior descending (LAD) artery, as confirmed by fractional flow reserve (FFR) assessment showing a value of more than 0.80.

**Fig. 4:**
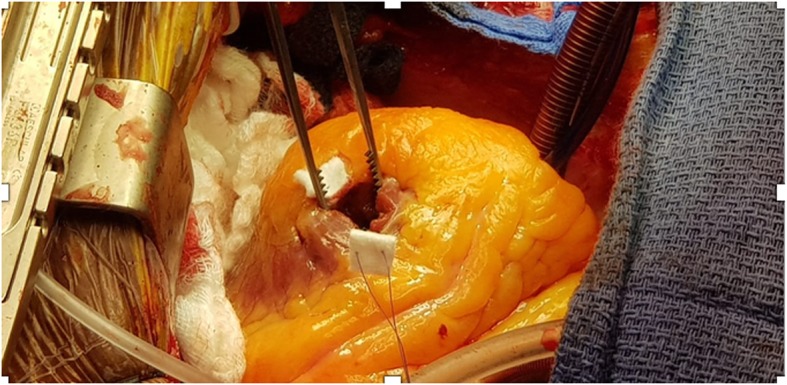
Operative view of ventriculotomy

**Fig. 5:**
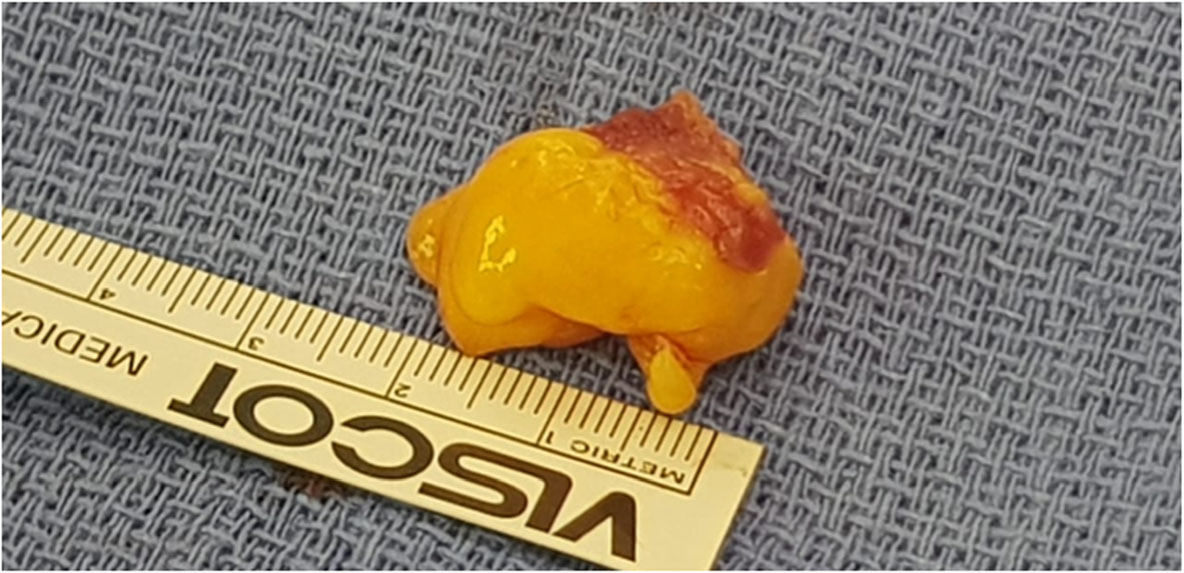
A mass of 2.5x1.5x1.5 cm in size was removed from LV

**Fig. 6:**
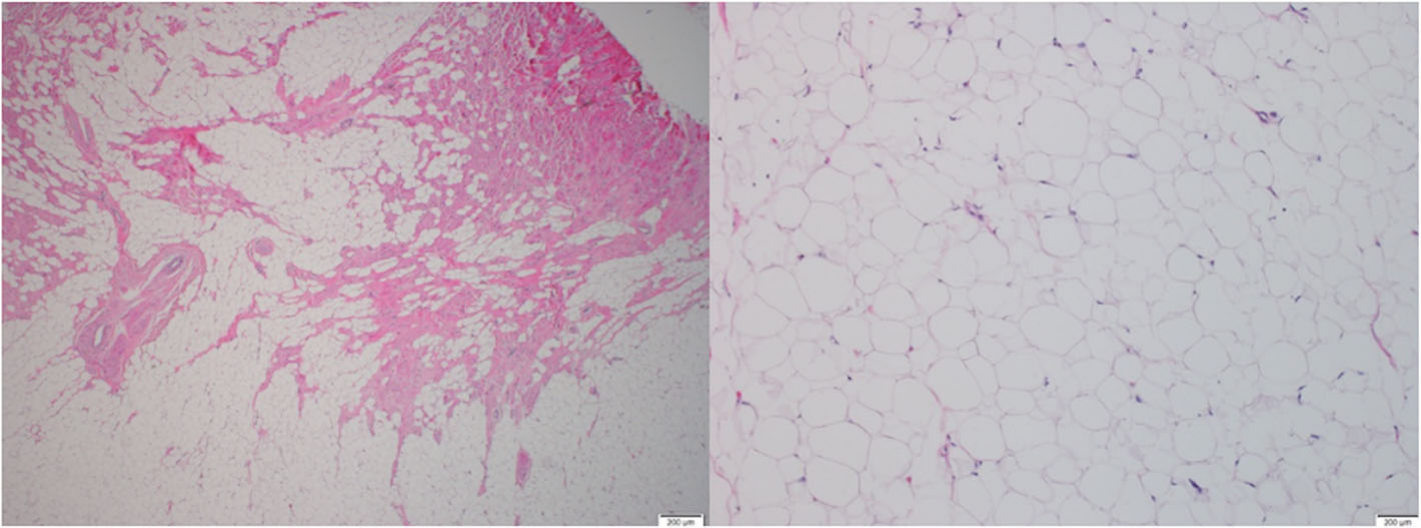
Histopathology of excised mass showed mature adipose cells, consistent with lipoma

Surgery was performed through a median sternotomy, with cardiopulmonary bypass. A left atriotomy approach was not able to visualize the stalk of the mass, so a left ventriculotomy was performed (Fig. 4) and the mass (2.5 × 1.5 × 1.5 cm) was removed (Fig. 5). No invasion into the ventricular myocardium was noted. The patient had an uneventful post-surgical course, and was discharged home. Histopathologic examination of the excised mass showed mature adipose cells, consistent with a lipoma (Fig. 6).

### Follow up

He presented with chest pain at 1 month from his surgery, and was found to have a moderate-sized pericardial effusion without evidence of cardiac tamponade. No signs of tumor recurrence were noted on echocardiogram. The patient was successfully- treated with Ibuprofen and Colchicine for post-pericardiotomy pericarditis.

## Discussion

Cardiac lipomas are rare benign primary cardiac tumors, accounting for 2–8% of all benign cardiac tumors [2, 3]. The typical ‘age at presentation’ of patients is between 40 and 60 years of age, but the presentation can occur at any age [4]. There is no gender predilection.

Cardiac lipomas are usually indolent and asymptomatic, particularly in the early stages. They are usually incidentally-discovered during cardiac investigations that are performed for other reasons. Symptoms such as dyspnea, pre-syncope, syncope or palpitations can occur if the tumor grows and causes LV inflow or outflow obstruction, LV dysfunction or invasion of the conduction system [2, 5–7]. Sudden cardiac death has been reported, but the true incidence is unknown given the rarity of this cardiac tumor [8–12]. The most common location for cardiac lipomas is the inter-atrial septum, followed by endocardium of RA and LV [3]. Other, less common, sites of involvement are the myocardium, sub-epicardium and pericardium [13]. On cardiac imaging, cardiac lipomas typical appear as a well-defined encapsulated mass [3]. This encapsulated appearance is what differentiates cardiac lipomas from lipomatous hypertrophy of the interatrial septum (LHIS) and adipo-sarcomas. Moreover, adipo-sarcomas have a tendency to invade the myocardium [2]. Echocardiography is the initial investigation of choice [3]. Subsequently, Cardiac Computed Tomography and CMR can be pursued to provide useful information regarding tissue characterization and the extent of myocardial infiltration [2, 3].

On CMR, lipomas have a homogeneous appearance of increased signal intensity on T1-weighted imaging, with a reduction in signal intensity in fat-saturated sequences [14]. Cardiac lipomas do not enhance with the administration of intravenous contrast. However, cardiac imaging fails to confirm the diagnosis in some cases, and surgical excision and histopathologic examination is required.

As the prevalence of cardiac lipomas is very low, there are no randomized clinical trials or large prospective cohorts to provide guidance or insight into the optimal treatment [4]. For large lesions that are causing obstruction, surgical resection is usually therapeutic and curative. Given the encapsulated nature of these tumors, they are not usually associated with embolization; and that is a rare indication for their surgical resection [3, 4, 15]. However, the general consensus is that surgical resection is commonly-pursued for all cardiac lipomas, regardless of symptoms or obstruction, due to the several reports in the literature of an associated risk of sudden cardiac death [8–12]. Cardiac lipomas are easy to resect as they are encapsulated and rarely invade the myocardium.

The surgical risk is low when the resection is performed early, when the lipoma is small and the LV function is preserved. The possible complications of lipoma resection include LV systolic dysfunction, ventricular septal defects and ventricular arrhythmias [16]. Death has also been reported after resection of a very large LV lipoma in a patient with pre-operative LV systolic dysfunction [2, 16]. In that particular case, the patient died within 2 weeks of surgery, due to refractory ventricular fibrillation and heart failure.

The usual surgical approach for lipoma resection is through a median sternotomy, after placing the patient on full cardiopulmonary bypass. However, the use of a thoracoscope–assisted limited sternotomy approach has been described [2]. In our case, ventriculotomy was performed as the access was restricted due to the apical location of the lipoma and the stalk being deep in a crypt.

The definitive diagnosis of cardiac lipomas is made on postoperative pathological examination. Lipomas are composed of mature fat cells that are surrounded by a fibrous membrane.

## Conclusion

The early diagnosis of LV lipoma is essential, and the treatment strategy should be individualized.

## Data Availability

Not applicable.

## Abbreviations

CMR: Cardiac Magnetic Resonance
ECG: Electrocardiogram
LHC: Left heart catheterization
LV: Left ventricle
RA: Right atrium
